# A Cross-Sectional Study Examining the Relationship Between Malnutrition and Gross Motor Function in Cerebral Palsy

**DOI:** 10.7759/cureus.55753

**Published:** 2024-03-07

**Authors:** Namrata Bharti, Ajeet K Dwivedi, Shikha Gupta, Abhishek K Singh, Bhoopendra Sharma, Imran Ahmed Khan

**Affiliations:** 1 Pediatrics, Baba Raghav Das Medical College, Gorakhpur, IND; 2 Physical Medicine and Rehabilitation, Baba Raghav Das Medical College, Gorakhpur, IND; 3 Community Medicine, Baba Raghav Das Medical College, Gorakhpur, IND

**Keywords:** muscle spasticity, motor disorders, malnutrition, growth disorders, cerebral palsy

## Abstract

Introduction

Cerebral palsy (CP) characterizes a range of permanent, nonprogressive symptoms of postural and motor dysfunction caused by an insult to the developing central nervous system in a fetus or an infant. CP manifests early in life, often within the first two to three years of age. CP is associated with poor growth, that is the deviation from the normal growth parameters. The prevalence of CP ranges from 2.0 to 3.5 per 1000 live births in high-income countries which is comparable to the estimates from low-income countries. Antenatal and perinatal insults are among the most commonly reported causes of CP; however, a large number of cases do not have an identifiable etiology of CP. The current study aims to examine the relationship between malnutrition and gross motor function in children with CP.

Materials and Methods

This study was conducted at the Department of Pediatrics and Neonatology, Nehru Hospital, Baba Raghav Das (BRD) Medical College, Gorakhpur (UP) over a period of one year (August 2020 to July 2021) after obtaining ethical clearance from the College Research Council. Children of age 1-15 years with CP attending the pediatric outpatient and inpatient departments were enrolled as the study participants after obtaining informed consent from a legal guardian. Assessment of motor function was done using the gross motor function classification system (GMFCS). Associations of malnutrition across levels of gross motor function were tested using Chi-square or Fisher’s exact test whichever was applicable. Statistical significance was set at p < 0.05 as significant. Data was analyzed using IBM SPSS Statistics for Windows, Version 21 (Released 2012; IBM Corp., Armonk, New York, United States).

Result

We analyzed 110 children with a diagnosis of CP (median age 6.5 years, interquartile range (IQR) 4.4-9.0 years). The majority (65/110; 59%) of the patients were male, and 68 (61.8%) delivered at term gestation. The most common presenting symptom among children with CP was seizures (79/110; 72.3%), the second most common being delayed milestones among 73 (66.8%), followed by difficulty in breathing among 63 (57.5%). The association between the anthropometric index of participants and GMFCS was found to be highly significant.

Conclusion

Most CP patients were facing gross motor disturbances. Spastic type of CP was most frequent, and more than half of the patients experienced feeding difficulty. A statistically significant association was found between gross motor functioning and the prevalence of malnutrition and stunting.

## Introduction

Cerebral palsy (CP) characterizes a range of permanent, nonprogressive symptoms of postural and motor dysfunction caused by an insult to the developing central nervous system in a fetus or an infant [1]. The motor disorders of CP are often accompanied by disturbances of sensation, cognition, communication, perception, and/or behavior and/or by a seizure disorder [2]. The prevalence of CP ranges from 2.0 to 3.5 per 1000 live births in high-income countries which is comparable to the estimates from low-income countries [3]. CP manifests early in life, often within the first two to three years of age. CP is a complex of symptoms rather than a specific disease. CP is the leading cause of childhood disability [4]. It is well recognized that CP is related to poor growth and the deviation from the routine growth parameter values increases with increasing levels of gross motor dysfunction [5,6]. A multidisciplinary team involving a dedicated pediatrician, pediatric neurologist, physiotherapist, occupational therapist, child psychologist, and social worker is needed to evaluate a child with CP. The pathophysiologic processes behind the majority of the CP symptoms are still poorly understood. Antenatal and perinatal insults are among the most commonly reported causes of CP; however, a large number of cases do not have identifiable etiology of CP [7]. Risk factors for CP may include but are not limited to low birth weight or preterm birth, multiple gestations, infertility treatments, infections during pregnancy, fever during pregnancy, Rh incompatibility, and exposure to toxic chemicals. Some maternal medical conditions like abnormal thyroid function, intellectual and developmental disability, seizures, complicated labor and delivery, jaundice, preterm or low birthweight, birth injury, etc., may also lead to CP. Malnutrition during the initial days of life may result in a delay in developmental milestones with early and remote consequences [8].

CP is a complex neuromuscular disorder known to impact gross motor function adversely [9,10]. Damage to the developing brain disrupts motor control and may generate spasticity, leading to diminished strength and aberrant musculoskeletal loading, further resulting in joint contractures and bone abnormalities. CP is nonprogressive in course, but secondary effects can continue and may worsen with maturation [11]. Children with CP and malnutrition are at increased risk of adverse outcome [12,13]. Malnutrition and growth impairment are associated both with nutritional and nonnutritional factors, including poor oral-motor function, gastric reflux, aspiration pneumonia, adverse neurotropic effects, and endocrine malfunctions [14,15]. Nutritional status and prognosis in children with CP are also found to be associated with socioeconomic factors. In low-income countries, poverty is a strong determinant which might aggravate the vulnerability of these children. The accurate assessment of nutritional status in children with CP is difficult. A reliable measurement of basic data such as weight, height, and body mass index (BMI) and their correct interpretation and analysis is required to identify children with nutritional risk and proper management. The present study was conducted to find out the association between malnutrition and gross motor function in children with CP using the gross motor function classification system (GMFCS).

## Materials and methods

Study design and study period

This is a tertiary care hospital-based cross-sectional study. The study was completed over a period of one year (August 2020 to July 2021).

Study center

This study was conducted at the Department of Pediatrics and Neonatology, Nehru Hospital, Baba Raghav Das (BRD) Medical College, Gorakhpur (UP). The study was approved by the College Research Council (CRC) of BRD Medical College, Gorakhpur, Uttar Pradesh, India.

Inclusion criteria and exclusion criteria

Children of age 1-15 years attending the pediatric outpatient department (OPD) and inpatient care with a diagnosis of CP contributed as the study participants. A written informed consent from the parents of the study participants was obtained beforehand.

Patients having a history of congenital malformations that would independently affect food intake, e.g., cleft lip, cleft palate, diagnosis associated with genetic syndromes, and spina bifida were excluded.

Sample size

The sample size was determined using the following formula: n = 4 pq/L2, where n is the required sample size, p is the estimated prevalence of CP at 3.8% (prevalence in India in 2017), q is 100-p, and L is the margin error at 5% (standard value of 0.05). The total calculated sample size was 110.

Study tools

Nutritional Assessment

Weight was measured on a digital scale in kilograms, with minimum cloths, or by calculating the difference between caretaker’s weight with and without the child. Height was measured in centimeters using a stadiometer, with the child in the standing or supine position, in those, who had no major skeletal deformities. In children with deformities, height was measured using the knee height equation, where height = (2.69 x knee height) + 24.2. Nutritional status and growth parameters were evaluated according to the WHO sex-specific weight-for-age, height-for-age, and weight-for-height growth charts. "Normal" will be considered when values had a z‐score between -1.99 and 1.99. Moderate undernutrition was established when z‐scores were between -2 and -2.99. Severe undernutrition was considered when z-scores were below -3.

Assessment of Motor Function

Based on the GMFCS, motor function was classified into five levels: level I, walks without limitations; level II, walks with limitations; level III, walks using a handheld mobility device; level IV, self-mobility with limitations, may use powered mobility; and level V, transported in a manual wheelchair) [16].

Statistical analysis

Categorical variables were presented as proportions and percentage. Age was presented as the median and interquartile range (IQR), and all the other continuous variables were presented as means and standard deviations. Normal distribution of all continuous variables was checked by skewness/kurtosis. Associations of malnutrition across levels of gross motor function were tested using Chi-square or Fisher’s exact test whichever was applicable. Statistical significance was set at p < 0.05 as significant. Data was analyzed using IBM SPSS Statistics for Windows, Version 21 (Released 2012; IBM Corp., Armonk, New York, United States).

## Results

We analyzed 110 children with a diagnosis of CP (median age 6.5 years, IQR 4.4-9.0 years). The majority (65/110; 59%) of the patients were male and 68 (61.8%) delivered at term gestation. Of the total 110 children with cerebral palsy, 73 (66.3%) were treated as in-patients and 37 (33.7%) were treated as out-patients, out of which 19 (17.27%) of them required PICU care and 13 (11.81%) of them required respiratory support. Most of the children belonged to the age group of 1 to 5 years with a mean age of 3.7 ± 1.4 years. Other characteristics of study participants are compiled in Table 1.

**Table 1:** Characteristics of participants (n = 110) n: number

Characteristics of participants		Number (%)
Gender	Male	65 (59)
	Female	45 (41)
Gestational age	Term	68 (61.8)
	Preterm	42 (38.2)
Type of patient	Inpatient	73 (66.3)
	Outpatient	37 (33.7)
Age group (month)	12-60	61 (55.4)
	61-120	38 (34.6)
	>120	11 (10)
Type of cerebral palsy	Spastic	73 (66.3)
	Dystonic	4 (3.6)
	Ataxic	7 (6.3)
	Hypotonic	13 (11.8)
	Mixed	13 (11.8)
Feeding difficulty	None	42 (38.1)
	Mild	39 (35.4)
	Moderate to severe	29 (26.3)
Gross motor function classification system) (GMFCS)	I	19 (17.2)
	II	7 (6.3)
	III	14 (12.7)
	IV	39 (35.2)
	V	31 (28.1)

The most common presenting symptom among children with cerebral palsy was seizures (79/110; 72.3%), the second most common being delayed milestones among 73 (66.8%), followed by difficulty in breathing among 63 (57.5%). Other common presenting symptoms are presented in Table 2.

**Table 2:** Presenting symptoms of participants with cerebral palsy (n = 110) n: number

Presenting symptoms of participants with cerebral palsy	Number	Percentage
Seizures	79	72.3
Developmental delay	73	66.8
Difficulty in breathing	63	57.5
Feeding difficulty	51	46.3
Cough	45	41.6
Fever	38	34.3
Abnormal body movements	32	29.3
Speech impairment	23	21.2
Hearing impairment	17	15.8
Others	8	7.3

Of the total 110 children, 22 (20%) were normal, 28 (25.45%) were moderately wasted, and 60 (54.54%) were severely wasted based on weight for height (Table 3).

**Table 3:** Distribution of participants with cerebral palsy according to weight for height (n = 110) n: number; SD: standard deviation

Characteristics of participants	Weight for height of participants		
	Normal (> -2SD)	Moderate wasting (< -2SD)	Severe wasting (< -3SD)
	n = 22	n = 28	n = 60
Types of cerebral palsy			
Spastic	13 (11.8%)	16 (14.5%)	44 (40.0%)
Ataxic	2 (1.8%)	2 (1.8%)	3 (2.7%)
Dystonic	1 (0.9%)	1 (0.9%)	2 (1.8%)
Hypotonic	3 (2.7%)	4 (3.6%)	6 (5.4%)
Mixed	3 (2.7%)	5 (4.5%)	5 (4.5%)
Sex			
Male	13 (11.8%)	17 (15.4%)	35 (31.8%)
Female	9 (8.1%)	11 (10.0%)	25 (22.7%)
Age group (month)			
12-60	14 (12.7%)	17 (15.4%)	30 (27.3%)
61-120	7 (6.4%)	7 (6.4%)	24 (21.8%)
>120	1 (0.9%)	4 (3.6%)	6 (5.4%)

Of the total 110 children, 29 (26.36%) were normal, 26 (23.63%) were moderately underweight, and 55 (50%) were severely underweight based on weight for age (Table 4).

**Table 4:** Distribution of participants with cerebral palsy according to weight for age n: number; SD: standard deviation

Characteristics of participants	Weight for age of participants		
	Normal (> -2SD)	Moderate underweight (< -2SD)	Severe underweight (< -3SD)
	n = 29	n = 26	n = 55
Types of cerebral palsy			
Spastic	16 (14.5%)	14 (12.7%)	43 (39.0%)
Ataxic	2 (1.8%)	3 (2.7%)	2 (1.8%)
Dystonic	2 (1.8%)	1 (0.9%)	1 (0.9%)
Hypotonic	4 (3.6%)	5 (4.5%)	4 (3.6%)
Mixed	5 (4.5%)	3 (2.7%)	5 (4.5%)
Sex			
Male	15 (13.6%)	14 (12.7%)	36 (32.7%)
Female	14 (12.7%)	12 (10.9%)	19 (17.2%)
Age group (month)			
12-60	18 (16.3%)	15 (13.6%)	28 (25.4%)
61-120	8 (7.3%)	10 (9.0%)	20 (18.2%)
>120	3 (2.7%)	1 (0.9%)	7 (6.4%)

Of the total 110 children, 26 (23.63%) were normal, 32 (29.09%) were moderately stunted, and 32 (47.27%) were severely stunted based on height for age (Table 5).

**Table 5:** Distribution of participants with cerebral palsy according to height for age n: number; SD: standard deviation

Characteristics of participants	Height for the age of participants		
	Normal (> -2SD)	Moderate stunting (< -2SD)	Severe stunting (< -3SD)
	n = 26	n = 32	n = 52
Types of cerebral palsy			
Spastic	14 (12.7%)	21 (19.0%)	38 (34.5%)
Ataxic	2 (1.8%)	1 (0.9%)	4 (3.6%)
Dystonic	1 (0.9%)	2 (1.8%)	1 (0.9%)
Hypotonic	5 (4.5%)	4 (3.6%)	4 (3.6%)
Mixed	4 (3.6%)	5 (4.5%)	4 (3.6%)
Sex			
Male	14 (12.7%)	19 (17.3%)	32 (28.8%)
Female	12 (10.9%)	13 (11.7%)	20 (18.0%)
Age group (month)			
12-60	17 (15.3%)	19 (17.1%)	25 (22.5%)
61-120	6 (5.4%)	12 (10.8%)	20 (18.0%)
>120	3 (2.7%)	1 (0.9%)	7 (6.3%)

The association between the anthropometric indices of the participants and GMFCS was highly significant (Table 6).

**Table 6:** Association between the nutritional status (WHO) and gross motor function GMFCS: gross motor function classification system; n: number; p-value, statistical significance; WHO, World Health Organization

Anthropometric index of participants		GMFCS of participants					
		I n = 19	II n = 7	III n = 14	IV n = 39	V n = 31	p-value
Weight for age							
Normal		6 (5.4%)	2 (1.8%)	5 (4.5%)	6 (5.4%)	10 (11.0%)	<0.002
Moderate underweight		4 (3.6%)	2 (1.8%)	4 (3.6%)	9 (8.1%)	7 (6.3%)	<0.001
Severe underweight		9 (8.1%)	3 (2.7%)	5 (4.5%)	24 (21.8%)	14 (12.7%)	<0.001
Height for age							
Normal	4 (3.6%)		1 (0.9%)	3 (2.7%)	11 (10.0%)	7 (6.3%)	<0.002
Moderate stunting	6 (5.4%)		1 (0.9%)	2 (1.8%)	12 (10.9%)	11 (10.0%)	<0.001
Severe stunting	9 (8.1%)		5 (4.5%)	9 (8.1%)	16 (14.5%)	13 (11.8%)	<0.001
Weight for height							
Normal	5 (4.5%)		1 (0.9%)	1 (0.9%)	9 (8.1%)	6 (5.4%)	<0.001
Moderate wasting	3 (2.7%)		2 (1.8%)	3 (2.7%)	11 (10.0%)	9 (8.1%)	<0.001
Severe wasting	11 (10.0%)		4 (3.6%)	10 (11.0%)	19 (17.2%)	16 (14.5%)	<0.001

## Discussion

This study was done to examine the association between malnutrition and gross motor function in children with CP based on the anthropometric assessment done according to growth charts by the WHO and motor assessment done according to the GMFCS. In our study, the majority of the patients were males (59%) with a male-to-female ratio of 1.4:1. Similarly, male predominance was also found in a study conducted by Bax et al. (1.6:1) [2]. Based on gestational age, 68 (61.8%) were term and 42 (38.2%) were preterm. We distributed children among three groups according to age 1-5 years, 5-10 years, 10-15 years 55.4%, 34.6%, and 10% in each group, respectively. Most of the children belonged to the age group of 1 to 5 years. The mean age was found to be 3.7 ± 1.4 years. In a study conducted by Singhi et al., the mean age was 3.1 ± 2.6 years (range 2 months to 16 years) [5].

Based on the types of CP, out of 110 children with CP, the most common among them was spastic 73 (66.3%), and the distribution among the rest was as follows, dystonic 4 (3.6%), ataxic 7 (6.3%), hypotonic 13 (11.8%), and mixed 13 (11.8%). A previous study by Brooks et al. found spastic dysfunction predominately and occurred in 79 of the 108 cases that completed the study. The other types (mixed n = 14; hypotonic n = 11; dyskinetic n = 3; and ataxic n = 1) occurred in 29 cases [13].

In our study, the most common presenting symptom among children with CP was seizures among 79 (72.3%) of the participants, while the second most common was delayed milestones among 73 (66.8%), followed by difficulty in breathing in 63 (57.5%); other common presenting symptoms were cough, fever, speech impairment, hearing impairment, and abnormal body movements. Pavone et al. also found epilepsy as one of the most frequent comorbidities of CP [17].

Anthropometry assessment is one of the important and well-established methods to screen malnutrition CP children. Assessing the nutritional status of CP children is highly essential. In our study, we found that more than half of children with CP were malnourished. Out of 110 children with CP, 54.5% were severely wasted, 50.0% were severely underweight, and 47.2% were severely stunted. A recent study by Kakooza et al. in 2015, however, reported that 52% of children with CP, who visited clinics, were malnourished [18]. In another study by Aydin, the prevalence of malnutrition was 57.2% based on the physicians' clinical judgment [19].

Of the total 110 children with CP, 17.2% were GMFCS level I, 6.3% were GMFCS level II, 12.7% were GMFCS level III, 35.4% were GMFCS level IV, and 28.1% were GMFCS level V. More than half of the children with CP were classified in functional levels IV and V. A cross‐sectional study from Colombia, Herrera‐Anaya et al. examined 177 children with a diagnosis of CP (median, 6.5 years; IQR, 4.4-9.0 years; 59.3% males) [20]. There were 70 (39.5%), 12 (6.8%), 10 (5.6%), 29 (16.4%), and 56 (31.6%) patients classified in levels I to V of the GMFCS, respectively, and a significantly higher prevalence of malnutrition among patients with lower motor function was found (p < 0.001) [20]. The proportion of children in levels IV and V in our study was higher as compared to some international studies [21, 22]. We also observed that the z‐scores of the anthropometric parameters, such as weight for age, height for age, and weight for height, progressively decreased as the levels of the GMFCS increased.

The observed associations may be attributed to the coexistence of gross motor and gastrointestinal dysfunctions in patients with CP. These gastrointestinal dysfunctions may include sucking difficulties, food refusal, sialorrhea, gastroesophageal reflux disease, oro-motor dysfunction, dysphagia, and constipation [23-25]. Children with malnutrition undergo a slowing down of organ systems owing to physiological and metabolic changes. [26] The presence of increased muscle tone, muscle spasms, and involuntary movements, which increase energy expenditure, might be the additional factors explaining this relationship.

Limitations

Although there were some limitations in our study, the results obtained using the anthropometric data demonstrate that the WHO gold standard may not be suitable for the anthropometric evaluation of children with CP. Using the same growth charts for children with neurological impairment tends to overestimate malnutrition and growth and development in children with CP. Furthermore, nutritional status was measured using the anthropometric measurements rather than the more precise ways, such as dual-energy X-ray absorptiometry. The small sample size is another limitation.

## Conclusions

The findings of this study revealed a high burden of malnutrition while also emphasizing the likelihood that malnutrition is overestimated in children with CP when the regular anthropometric assessment was done using the growth charts for the general pediatric population. Spastic type of CP was most frequent, and more than half of the patients experienced feeding difficulty. Most CP patients were facing gross motor disturbances. A statistically significant association was found between gross motor functioning and the prevalence of malnutrition and stunting. These findings emphasize the critical importance of early detection and intervention for malnutrition among children with CP, necessitating a multidisciplinary approach involving pediatricians, nutritionists, and therapists. Future research should focus on developing and validating specific nutritional screening tools tailored to the unique needs of children with CP and conducting longitudinal studies to understand the relationship between gross motor function and nutritional status.

## References

[1] Vova J (2022). Cerebral palsy: an overview of etiology, types and comorbidities. OBM Neurobiol.

[2] Patel DR, Neelakantan M, Pandher K, Merrick J (2020). Cerebral palsy in children: a clinical overview. Transl Pediatr.

[3] Gladstone M (2010). A review of the incidence and prevalence, types and aetiology of childhood cerebral palsy in resource-poor settings. Ann Trop Paediatr.

[4] Sewell MD, Eastwood DM, Wimalasundera N (2014). Managing common symptoms of cerebral palsy in children. BMJ.

[5] Singhi PD, Ray M, Suri G (2002). Clinical spectrum of cerebral palsy in north India--an analysis of 1,000 cases. J Trop Pediatr.

[6] Oskoui M, Coutinho F, Dykeman J, Jetté N, Pringsheim T (2013). An update on the prevalence of cerebral palsy: a systematic review and meta-analysis. Dev Med Child Neurol.

[7] Jin SC, Lewis SA, Bakhtiari S (2020). Mutations disrupting neuritogenesis genes confer risk for cerebral palsy. Nat Genet.

[8] Verma GK, Chand R, Khan IA, Kumar A, Yadav RK (2023). Association of developmental milestones with vitamin B12 and folate status among hospitalized severe acute malnutrition children at a tertiary care center in North India. MGM Journal of Medical Sciences.

[9] Lee BH (2017). Relationship between gross motor function and the function, activity and participation components of the International Classification of Functioning in children with spastic cerebral palsy. J Phys Ther Sci.

[10] Roldan A, Sarabia JM, Gómez-Marcos G, Reina R (2020). An observational tool to assess activity limitation in ambulatory people with cerebral palsy when performing motor skills. Int J Environ Res Public Health.

[11] MacWilliams BA, Prasad S, Shuckra AL, Schwartz MH (2022). Causal factors affecting gross motor function in children diagnosed with cerebral palsy. PLoS One.

[12] Hurvitz EA, Green LB, Hornyak JE, Khurana SR, Koch LG (2008). Body mass index measures in children with cerebral palsy related to gross motor function classification: a clinic-based study. Am J Phys Med Rehabil.

[13] Brooks J, Day S, Shavelle R, Strauss D (2011). Low weight, morbidity, and mortality in children with cerebral palsy: new clinical growth charts. Pediatrics.

[14] Finbråten AK, Martins C, Andersen GL (2015). Assessment of body composition in children with cerebral palsy: a cross-sectional study in Norway. Dev Med Child Neurol.

[15] Calis EA, Veugelers R, Sheppard JJ, Tibboel D, Evenhuis HM, Penning C (2008). Dysphagia in children with severe generalized cerebral palsy and intellectual disability. Dev Med Child Neurol.

[16] Palisano R, Rosenbaum P, Walter S, Russell D, Wood E, Galuppi B (1997). Development and reliability of a system to classify gross motor function in children with cerebral palsy. Dev Med Child Neurol.

[17] Pavone P, Gulizia C, Le Pira A (2020). Cerebral palsy and epilepsy in children: clinical perspectives on a common comorbidity. Children (Basel).

[18] Kakooza-Mwesige A, Tumwine JK, Eliasson AC, Namusoke HK, Forssberg H (2015). Malnutrition is common in Ugandan children with cerebral palsy, particularly those over the age of five and those who had neonatal complications. Acta Paediatr.

[19] Aydin K (2018). A multicenter cross-sectional study to evaluate the clinical characteristics and nutritional status of children with cerebral palsy. Clin Nutr ESPEN.

[20] Herrera-Anaya E, Angarita-Fonseca A, Herrera-Galindo VM, Martínez-Marín RD, Rodríguez-Bayona CN (2016). Association between gross motor function and nutritional status in children with cerebral palsy: a cross-sectional study from Colombia. Dev Med Child Neurol.

[21] Ruiz Brunner ML, Cieri ME, Rodriguez Marco MP, Schroeder AS, Cuestas E (2020). Nutritional status of children with cerebral palsy attending rehabilitation centers. Dev Med Child Neurol.

[22] Burgess A, Reedman S, Chatfield MD, Ware RS, Sakzewski L, Boyd RN (2022). Development of gross motor capacity and mobility performance in children with cerebral palsy: a longitudinal study. Dev Med Child Neurol.

[23] Campanozzi A, Capano G, Miele E (2007). Impact of malnutrition on gastrointestinal disorders and gross motor abilities in children with cerebral palsy. Brain Dev.

[24] Badaru UM, Umar AL, Abdullahi A, Usman JS, Ogwumike OO (2023). Influence of malnutrition and body composition on the gross motor function of children with cerebral palsy in Kano, Nigeria: a cross-sectional study. Bulletin Facul Phys Ther.

[25] Zhao Y, Tang H, Peng T (2023). Relationship between nutritional status and severity of cerebral palsy: a multicentre cross-sectional study. J Rehabil Med.

[26] Verma GK, Yadv RK, Chand R, Khan IA, Katiyar SB, Singh M (2022). Prognostic significance of serum biochemistry profile in children with severe acute malnutrition. Cureus.

